# Grafting of bovine serum albumin proteins on plasma-modified polymers for potential application in tissue engineering

**DOI:** 10.1186/1556-276X-9-161

**Published:** 2014-04-04

**Authors:** Nikola Slepičková Kasálková, Petr Slepička, Zdeňka Kolská, Petra Hodačová, Štěpánka Kučková, Václav Švorčík

**Affiliations:** 1 Department of Solid State Engineering, Institute of Chemical Technology Prague, Technicka 5, Prague 166 28, Czech Republic; 2 Faculty of Science, J. E. Purkyne University, Ceske Mladeze 8, Usti nad Labem 400 96, Czech Republic; 3 Department of Biochemistry and Microbiology, Institute of Chemical Technology Prague, Technicka 5, Prague 166 28, Czech Republic

**Keywords:** Polymers, Plasma treatments, Protein grafting, Surface characterization, Cell interaction

## Abstract

In this work, an influence of bovine serum albumin proteins grafting on the surface properties of plasma-treated polyethylene and poly-l-lactic acid was studied. The interaction of the vascular smooth muscle cells with the modified polymer surface was determined. The surface properties were characterized by X-ray photoelectron spectroscopy, atomic force microscopy, nano-LC-ESI-Q-TOF mass spectrometry, electrokinetic analysis, and goniometry. One of the motivations for this work is the idea that by the interaction of the cell with substrate surface, the proteins will form an interlayer between the cell and the substrate. It was proven that when interacting with the plasma-treated high-density polyethylene and poly-l-lactic acid, the bovine serum albumin protein is grafted on the polymer surface. Since the proteins are bonded to the substrate surface, they can stimulate cell adhesion and proliferation.

## Background

Tissue engineering (TE) is the discipline which includes both creation of the new tissue and design and realization of the cells on substrates [1,2]. Substrates play a key role in creation of the cell environment [3]. To guide the organization, growth, and differentiation of cells in TE constructs, the biomaterial scaffold should be able to provide not only a physical support but also the chemical and biological clues needed in forming functional tissue [4-6].

Biomaterials and various synthetic and natural materials, such as polymers, ceramics, metals, or their composites, have been investigated and used in different manners [5,7]. Polymeric materials have been widely studied as substrates for tissue engineering due to their unique features such as mechanical properties, high availability, low cost, and relatively easy design and production [6,8]. However, only a few polymers provide the biocompatibility needed to be used with the cells *in vitro* and *in vivo*[9]. High-density polyethylene (HDPE) has been extensively used for application such as the part of orthopedic implants [10]. To induce a regeneration process and to avoid the problems due to tissue replacement with a permanent implant, research has been oriented towards the development of polymers that would degrade and could be replaced by human tissue produced by the cells surrounding the material [9]. Despite of their advantages, however, some of their characteristic properties, like wettability, adhesion, surface composition, and suchlike are insufficient for many applications. The positive effect of the above-mentioned properties and also biocompatibility of the polymer surface provide an opportunity of modification of existing material with bioactive molecules (amino acids, peptides, anticoagulants) bound by covalent bonds to polymer surface [11-13].

Polymer surfaces are often modified by thin layers of protein-like collagen or fibronectin to improve their biocompatibility [14]. Bioactive molecules influence also the growth factors and regulate cell adhesion, migration, and proliferation [9,15]. Bovine serum albumin (BSA) is a globular protein that is used in numerous biochemical applications. Bovine serum albumin (BSA) can be used as a reference (model) protein in which its properties are compared with other proteins. BSA is also included in the protein part of the various media used for operations with cells. BSA was chosen as a representative protein present in cell culture as a supplement to increase the growth and productivity of cells and increase overall cell health.

A very important part of the general study of biocompatibility of materials is the surface characterization of the prepared substrates and adhered bioactive compounds. As basic parameters influencing the cell-substrate interaction, surface chemistry, polarity, wettability morphology, and roughness can be included.

In this work, the influence of BSA protein grafting on the surface properties of the polyethylene (HDPE) and poly-l-lactide acid (PLLA) was studied. HDPE was chosen as the representative of the non-polar/non-biodegradable polymer. With its very simple structure containing only carbon and hydrogen atoms, this polymer can serve as a model material. PLLA was chosen as a polar/biodegradable polymer, whose cell affinity is often compromised because of its hydrophobicity and low surface energy [16]. The surface properties were characterized by X-ray photoelectron spectroscopy, nano-LC-ESI-Q-TOF mass spectrometry, atomic force microscopy, electrokinetic analysis, and goniometry. One of the motivations for this work is the idea that due to cell interaction with the substrate, the proteins will form an interlayer between the cell and the substrate surface [17].

## Methods

### Materials and chemical modification

The experiments were performed on HDPE foil (thickness 40 μm, density 0.951 g cm^−3^, Granitol a.s. CR, Moravský Beroun, Czech Republic) and biopolymer PLLA foil (50 μm, 1.25 g cm^−3^, Goodfellow Ltd., Huntingdon, UK).

The surface modification of polymer substrates consisted of plasma treatment and subsequent grafting with proteins. The samples were modified by plasma discharge on Balzers SCD 050 device (BalTec Maschinenbau AG, Pfäffikon, Switzerland). The parameters of the deposition were DC Ar plasma, gas purity 99.995%, flow 0.3 l s^−1^, pressure 8 Pa, power 3 W, electrode distance of 50 mm, and time 300 s.

Immediately after treatment, the activated polymer surface was grafted by immersion into water solution of BSA (concentration 2 wt.%, Sigma-Aldrich Corporation, St. Louis, MO, USA) for 24 h at room temperature (RT). The excess of non-bound molecules was removed by consequent immersion of the samples into distilled water for 24 h. The samples were dried at RT for 13 h.

### Diagnostic techniques

The surface wettability was determined by water contact angle (WCA) measurement immediately after modification and after 17 days using distilled water (drop of volume 8 μl) at 20 different positions and surface energy evaluation system (Advex Instruments, Brno, Czech Republic). WCA of the plasma-treated samples strongly depends on the time from treatment.

The presence of the grafted protein molecules on the modified surface was detected by nano-LC-ESI-Q-TOF mass spectrometry. The samples were placed in Petri dish, and 10 μl of solutions (2 μl trypsin, concentration 20 μg μl^−1^ in 100 μl 50 mmol l^−1^ NH_4_HCO_3_) was applied on the sample surface. In the inside perimeter of Petri dishes, pieces of wet pulp were placed, in order to avoid drying of the solution on the surface of foils, and consequently the dish was closed. After 2 h of the molecule cleavage, new peptides were concentrated and desalted by reverse-phase zip-tip C18 (EMD Millipore Corporation, Billerica, MA, USA) at RT.

The presence of the carbon, oxygen, and nitrogen atoms in the modified surface layer was detected by X-ray photoelectron spectroscopy (XPS). The spectra of samples were measured with Omicron Nanotechnology ESCAProbeP spectrometer (Omicron Nanotechnology GmbH, Taunusstein, Germany) (1,486.7 eV, step size 0.05 eV, area 2 × 3 mm^2^). This elemental analysis was performed 17 days after modification of the samples.

The changes in surface morphology and roughness of samples were examined 17 days after modification by atomic force microscopy (AFM) using a Veeco CP II device (Bruker Corporation CP-II, Santa Barbara, CA, USA) (‘tapping’ mode, probe RTESPA-CP, spring constant 20 to 80 N∙m^−1^). The surface roughness value (*R*_a_) represents the arithmetic average of the deviation from the center plane of the samples.

The electrokinetic analysis (zeta potential) of the samples was done using SurPASS instrument (Anton Paar, Graz, Austria), (adjustable gap cell, 0.001 mol∙dm^−3^ electrolyte KCl, pH = 6.3, RT). The values of the zeta potential were determined by two methods, a streaming current and a streaming potential and calculated by Helmholtz-Smoluchowski and Fairbrother-Mastins equations [18]. Each sample was measured four times with the experimental error of 10%.

### Biological test of adhesion and proliferation

For evaluation of cell number and morphology in cell culture experiments, three pristine and modified HDPE and PLLA samples were used for analysis by randomly chosen fields. The samples were sterilized for 1 h with 70% ethanol, air-dried in a sterile environment to prevent possible negative effects of alcohol on the cells, and inserted into 12-well plates (TPP, well diameter 2 mm). Samples were seeded with smooth vascular muscle cells (VSMCs) derived from rat aorta by an explantation method (passage 7). VSMCs were seeded with the density 17,000 cells/cm^2^ into 3 ml of Dulbecco's modified Eagle's minimum essential medium (DMEM, Sigma) supplement with 10% fetal bovine serum (FBS, Sebak GmbH, Aidenbach, Germany). The cells were cultivated for 2, 4, and 6 days at 37°C in a humidified air atmosphere containing 5% CO_2_.

On the 2nd, 4th, and 6th day after seeding, the cells were rinsed in phosphate buffered saline (PBS) and fixed for 1 h in 70% cold ethanol (−20°C). The samples used for analysis by randomly chosen field were stained for 40 min with a combination of fluorescent membrane dye Texas Red C_2_-maleimide (Molecular probes, Invitrogen, Carlsbad, CA, USA) and a nuclear dye Hoechst no 33342 (Sigma). The number, morphology, and distribution of cells on substrate surface were then evaluated on photographs taken under an Olympus I×51 microscope using an Olympus DP 70 digital camera (Olympus America Inc., Center Valley, PA, USA). The number of cells was determined using image analysis software NIS Elements (Nikon Instruments Inc., Melville, NY, USA).

## Results and discussion

### Physical and chemical properties

**Figure 1:**
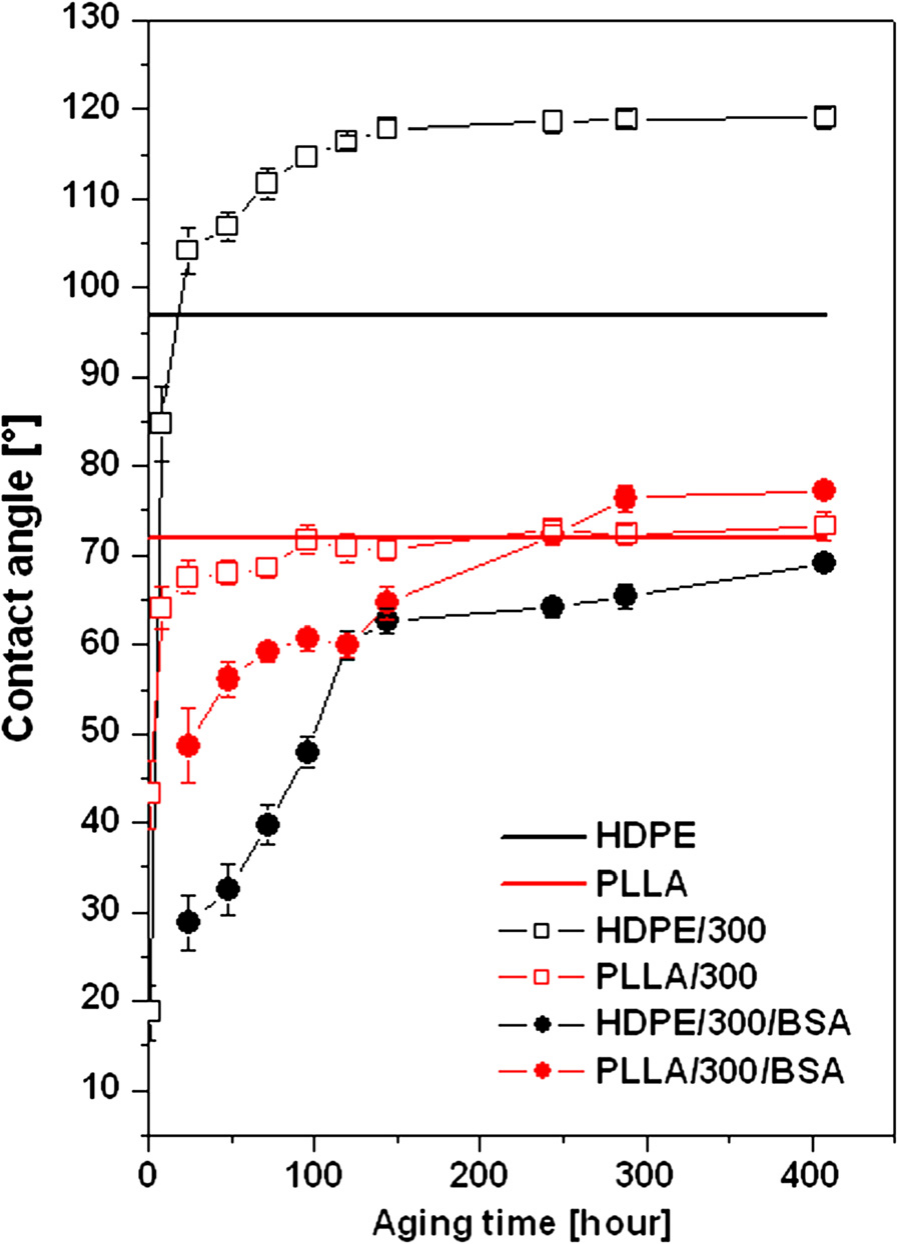
Dependence of WCA of pristine, plasma-treated and subsequently grafted polymers on the aging time.

Figure 1 represents the dependence of the WCA of pristine, plasma-treated, and subsequently grafted samples on the aging time (time from treatment). It is evident that immediately after plasma treatment (1 h), WCA decreases sharply to the minimal value which means the increasing the surface wettability. This effect corresponds with oxidation of the surface layer caused by creation of new polar groups [19]. Further, WCA increases with the increasing aging time, which can be explained by the rearrangement of the newly created functional polar groups of the macromolecular chains into the polymer bulk [19]. The saturated value of WCA of plasma-treated HDPE is higher than value of pristine HDPE, while at PLLA it is near the value of pristine PLLA. The time needed for the stabilization of the surface layer (for aging of the polymer) is 144 h for HDPE and 96 h for PLLA. From Figure 1, it is evident that immediately after the protein grafting, the samples have higher values of WCA in comparison with only plasma-treated samples. The value of WCA of grafted HDPE increases for the first 120 h faster than values measured on grafted PLLA. After reaching this time, the WCA value of grafted HDPE is not significantly changed and remains significantly lower than pristine or aged treated HDPE. The WCA of grafted PLLA is stabilized after approximately 244 h on the value higher than that of pristine or treated PLLA.

The presence of grafted protein on modified samples was proved using mass spectrometry. First five (for HDPE) or four (for PLLA) peptides detected on the grafted HDPE and PLLA, respectively, are shown in Table 1. The protein that was identified by the largest number of peptides was BSA in both cases, as expected. Furthermore, Table 1 includes other analyzed proteins which come from the cattle (cow, *Bos taurus*) and sheep (*Ovis aries*) that have been identified at least with nine peptides. The other found proteins come from probably commercially supplied BSA (purity 96%). Although the samples were grafted with BSA and therefore proteins from other species would not appear on the surface of samples, it is possible to explain their identification on the basis of similar amino acid sequences between even-toed ungulate (artiodactyls).

**Table 1:** Peptides detected on the surface of grafted HDPE and PLLA proved using mass spectrometry

**Sample**	**Accession**	**Protein**	**M**_**w **_**(kDa)**	**Peptides**
HDPE	ALBU BOVIN	Serum albumin	69.2	21
	FIBA BOVIN	Fibrinogen alpha chain	67.0	11
	APOA1 BOVIN	Apolipoprotein A-I	30.3	15
	CERU SHEEP	Ceruloplasmin	119.1	11
	ALBU_SHEEP	Serum albumin	69.1	11
PLLA	ALBU_BOVIN	Serum albumin	69.2	21
	CERU_SHEEP	Ceruloplasmin	119.1	11
	FIBA_BOVIN	Fibrinogen alpha chain	67.0	9
	APOA1_BOVIN	Apolipoprotein A-I	30.3	10

Detected peptides grafted on the HDPE and PLLA surfaces proved using mass spectrometry. The first five peptides were detected on HDPE and four on PLLA.

The atomic concentrations of the carbon, oxygen, and nitrogen in the polymer surface layer of pristine, plasma-treated, and grafted samples are summarized in Table 2. The presence of oxygen was detected on the surface of plasma-modified HDPE, which confirms previous findings and assumption that plasma treatment leads to oxidation of the surface layer due to creation of oxygen-containing polar groups [19]. In the case of treated PLLA, a slight reduction of oxygen in modified layers was detected. The minimum quantity of nitrogen present on plasma-treated samples was caused by reaction of activated samples with air atmosphere. The surface layers of substrates grafted by BSA contained comparable concentration of nitrogen and oxygen confirming BSA grafting. These results are in agreement with determination of contact angle.

**Table 2:** Atomic concentration of selected elements determined in surface layer of polymers using XPS

**Substrate**	**Treatment (s)**	**Atomic concentration (%)**		
		**C**	**O**	**N**
HDPE	0	100.0	-	-
	300	81.8	16.8	1.4
	300/BSA	67.9	18.1	14.0
PLLA	0	63.6	36.4	-
	300	65.2	33.3	1.5
	300/BSA	69.4	17.2	13.4

The atomic concentration of the carbon (C(1 *s*)), oxygen (O(1 *s*)), and nitrogen (N(1 *s*)) in the HDPE and PLLA surface layers of pristine (0), plasma-treated for 300 s (300), and BSA-grafted (300/BSA) was determined by XPS.

**Figure 2:**
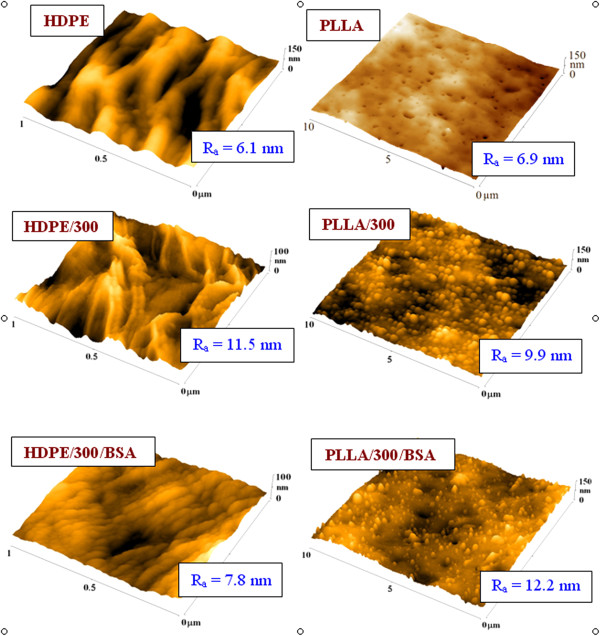
**AFM images and surface roughness *R***_**a **_**of pristine, plasma-treated, and subsequently grafted samples of polymer foils.**

The surface morphology and roughness of the samples were examined by AFM. From the scans shown in Figure 2, it is evident that the treatment of foils leads to an increase of surface roughness. This can be caused by a different ablation rate of crystalline and amorphous phase [19]. It is also evident that in the case of HDPE, the plasma treatment caused the highlight of the lamellar structure and in the case of PLLA, it resulted in the creation of granular structure. The subsequent grafting by the BSA leads to different surface arrangements of both polymers. The lamellar structure of HDPE is maintained, but it is noticeably lower and finer in comparison with plasma-treated one and the surface roughness considerably decreased. In the case of grafted PLLA, the granular morphology is maintained but the ‘tops’ are sharper and narrower than only plasma-treated one and the surface roughness increased.

**Figure 3:**
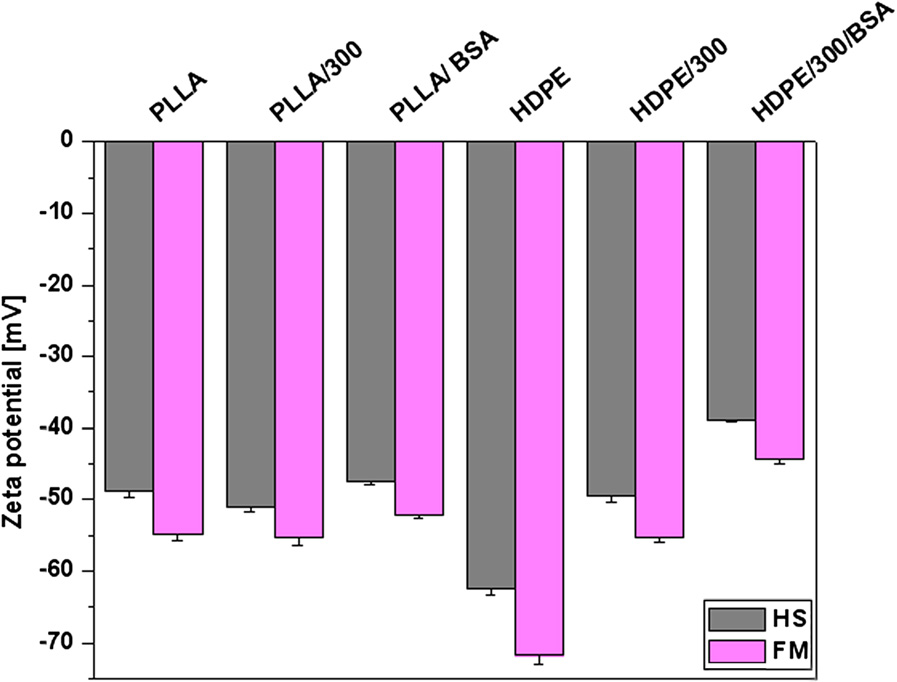
**Zeta potential of pristine, plasma-treated, and subsequently grafted samples of polymer foils.** The value was determined by Helmholtz-Smoluchowski (HS) and Fairbrother-Mastins (FM) equations.

The zeta potential (ZP) of all samples is shown in Figure 3. It is evident that pristine PLLA is polar in comparison with pristine HDPE. It corresponds very well with the contact angle measurement (Figure 1). The modifications of PLLA do not play an important role on ZP, while changes in ZP at HDPE are more significant. After plasma treatment of HDPE, ZP increases which indicates much polar surface is caused by the presence of oxygen polar groups. These results are in comparison with XPS measurement (Table 2). The increase of ZP at HDPE is also caused by grafting of BSA due to the presence of nitrogen on the surface. The slight increase of ZP after grafting of BSA has been also obtained at PLLA but not too significant. The differences between ZP obtained by both of applied methods (HS and FM) at individual samples indicate the different *R*_a_. This difference (Figure 3) is higher at HDPE, which indicates higher *R*_a_ in comparison with PLLA (Figure 2).

### Cell adhesion, growth, and proliferation

**Figure 4:**
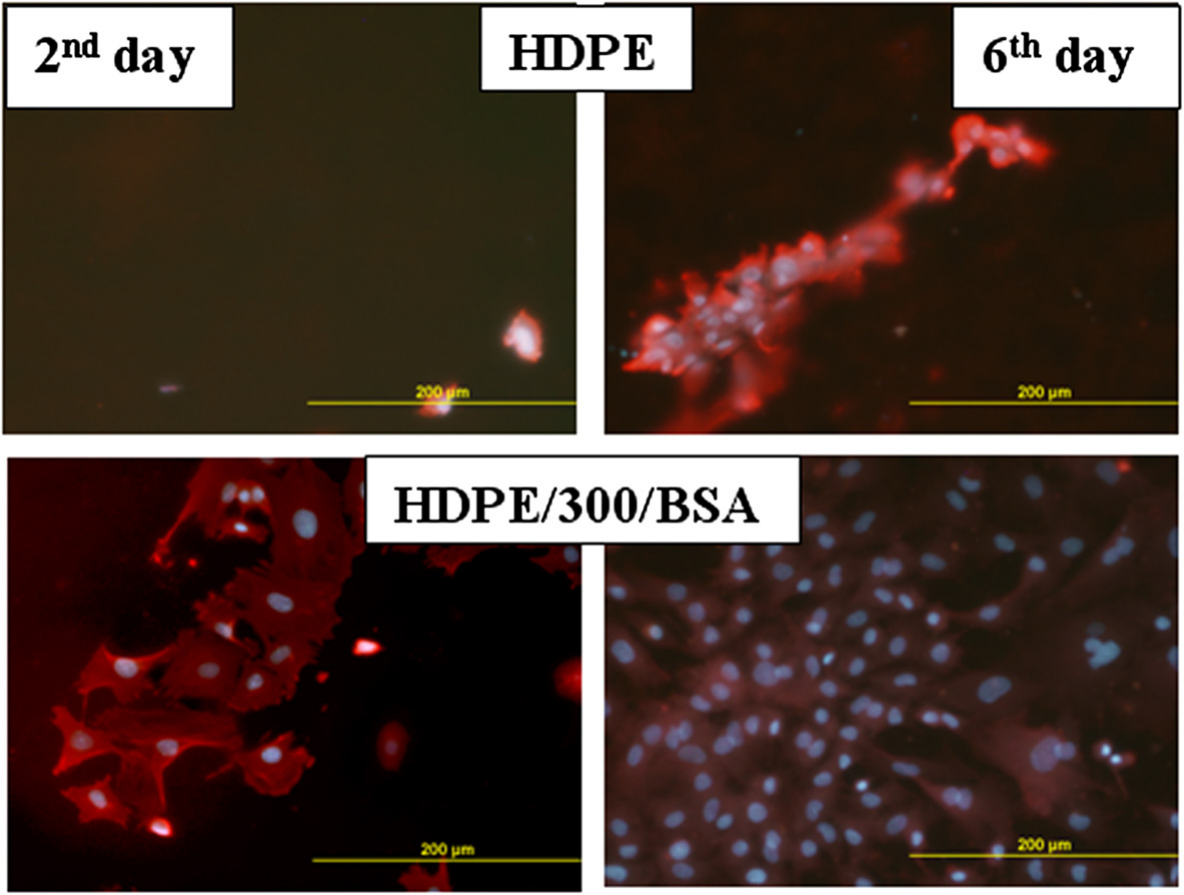
Photographs of VSMCs cultivated on pristine and BSA-grafted HDPE for 2 and 6 days.

Numbers of the cultivated VSMCs on the pristine and BSA-grafted HDPE and PLLA for 2, 4, and 6 days after seeding are shown in Table 3. On the 2nd day after seeding, the number of the VSMCs was significantly lower on the pristine HDPE in comparison with HDPE grafted by BSA. From the 2nd to the 4th day after seeding, the intense increase of VSMCs on the grafted HDPE was detected. On the contrary, the number of cells cultivated 4 days from seeding on the pristine HDPE was comparable with the 2nd day. Between the 4th and 6th day, the cell's proliferation on the grafted HDPE slowed down, probably due to reaching the cell's confluence. In the case of pristine HDPE, from the 4th to 6th day, the VSMCs started to proliferate and after 6 days of cultivation, they reached the number *ca* 22,000 cells/cm^2^, which is considerably less than the number of cells on grafted HDPE (*ca* 85,200 cells/cm^2^). The cells cultivated on the grafted HDPE were better spread; spreading areas were larger in comparison to pristine. After 6 days of cultivation, the cells cover homogeneously the surface of the grafted HDPE (Figure 4).

**Table 3:** **Number of VSMCs (cells/cm**^**2**^**) cultivated 2, 4, and 6 days on HDPE and PLLA**

**Substrate**	**Number of VSMCs (cells/cm**^**2**^**) cultivated**		
	**2 days**	**4 days**	**6 days**
HDPE	2,342	4,698	26,146
HDPE/300/BSA	18,268	73,169	85,234
PLLA	8,623	70,675	102,164
PLLA/300/BSA	12,662	85,225	129,681

Number of the VSMCs (cells/cm^2^) cultivated 2, 4, and 6 days on the pristine and BSA-grafted HDPE and PLLA of pristine (HDPE or PLLA), plasma-treated for 300 s, and BSA-grafted (/300/BSA).

**Figure 5:**
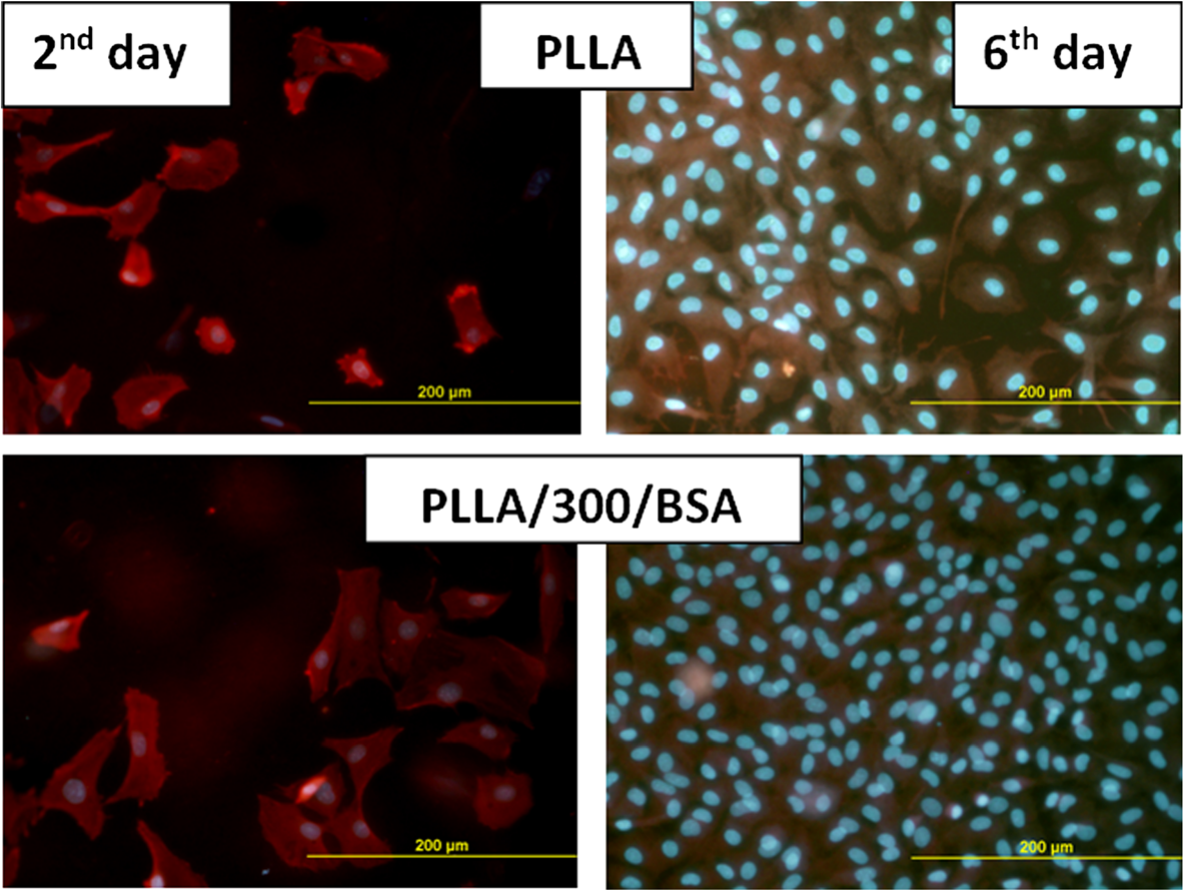
Photographs of VSMCs cultivated on pristine and BSA-grafted PLLA for 2 and 6 days.

The number of cells cultivated on the pristine and grafted PLLA was higher in comparison with pristine and grafted HDPE for 2, 4, and 6 days from seeding. The cells were better spread on PLLA after 2 days in comparison with HDPE. The entire surface of PLLA grafted sample was homogeneously and densely covered with confluent layer of VSMCs after 6 days of cultivation (see Figure 5).

The explanation of biocompatibility improvement of surface after plasma modification and protein grafting is connected with surface chemistry change, especially with amino groups presented on the modified surface. It is known that the major proteins (especially proteins of fetal bovine serum) as well as cell membranes are negatively charged under physiological pH. The adhesion of cells with negatively charged membranes may be facilitated by electrostatic interactions and the better cell adhesion may be expected on positively charged surfaces [20-22]. The surface charge (of solid substrates and of cells) significantly determines both cell-cell and cell-solid interactions. In low ionic strength environment, the adhesion is influenced mostly by electrostatic interactions between surfaces, where the surface chemistry, surface functional groups, and surface charge play the important role; while in increasing ionic strength (increasing concentration of surroundings), the importance of non-polar (hydrophobic) interactions grows [23]. Also, it was presented earlier for human umbilical vein endothelial cells [24] or for human fibroblasts [25] that better protein adsorption occurs if the surface contains -NH_2_ groups. Adsorbed proteins play a major role in the attachment of anchorage-dependent cells through their binding to integrins [25].

These results are contrary to the majority of theories, in which albumin is considered a non-adhesive molecule. But albumin can support of the adsorption of some molecules (like vitronectin or fibronectin) from the culture serum and thus can indirectly and positively influence cell's adhesion and proliferation. The molecules may be synthesized and deposited by VSMCs and may cause the increase of the cell's activity [26].

## Conclusions

It was proven that during interaction of BSA protein with the plasma-treated polyethylene and poly-l-lactic acid, BSA protein is grafted on their surfaces. Chemically bonded BSA protein was confirmed by XPS, mass spectrometry, AFM, electrokinetic analysis, and goniometry. This result is a significant contribution to the understanding of cell and substrate behavior during cell interaction with chemically active polymer in tissue engineering field. Due to plasma treatment and subsequent BSA grafting to polymer surface, the cell adhesion and proliferation can be stimulated due to the presence of active functional groups on the surface, which improves the electrostatic interactions between substrates and cells.

## Abbreviations

AFM: atomic force microscopy; BSA: bovine serum albumin; FM: Fairbrother-Mastins equation; HDPE: high-density polyethylene; HS: Helmholtz-Smoluchowski equation; PLLA: poly-l-lactic acid; RT: room temperature; TE: tissue engineering; VSMC: vascular smooth muscle cell; WCA: water contact angle; XPS: X-ray photoelectron spectroscopy; ZP: zeta potential.

## Competing interest

The authors declare that they have no competing interests.

## Authors’ contributions

NSK carried out the sample preparation, determined the contact angle, performed the biological tests, and participated in writing the article. PS analyzed the surface morphology, evaluated the surface roughness, and wrote some paragraphs of the article regarding AFM analysis, and participated on the paper corrections. ZK analyzed the zeta potential of the pristine and modified samples. PH and ŠK performed analysis and evaluation of the mass spectrometry. VŠ participated in the study coordination and paper corrections. All authors read and approved the final manuscript.
